# Oral vaccination with a recombinant *Lactobacillus plantarum* expressing the *Eimeria tenella* rhoptry neck 2 protein elicits protective immunity in broiler chickens infected with *Eimeria tenella*

**DOI:** 10.1186/s13071-024-06355-w

**Published:** 2024-06-28

**Authors:** Tongxuan Zhang, Hangfan Qu, Wei Zheng, Yanan Zhang, Yanning Li, Tianxu Pan, Junyi Li, Wentao Yang, Xin Cao, Yanlong Jiang, Jianzhong Wang, Yan Zeng, Chunwei Shi, Haibin Huang, Chunfeng Wang, Guilian Yang, Jingwei Zhang, Nan Wang

**Affiliations:** 1 College of Veterinary Medicine, Jilin Agricultural University, Changchun, 130118 China; 2 Jilin Provincial Key Laboratory of Animal Microecology and Healthy Breeding, Jilin Agricultural University, Changchun, 130118 China; 3 Engineering Research Center of Microecological Vaccines (Drugs) for Major Animal Diseases, Ministry of Education, Jilin Agricultural University, Changchun, 130118 China; 4 College of Foreign Languages, Jilin Agricultural University, Changchun, 130118 China

**Keywords:** *Eimeria tenella*, *Lactobacillus plantarum*, RON2, Dcpep, Vaccine

## Abstract

**Background:**

Chicken coccidiosis is a protozoan disease that leads to considerable economic losses in the poultry industry. Live oocyst vaccination is currently the most effective measure for the prevention of coccidiosis. However, it provides limited protection with several drawbacks, such as poor immunological protection and potential reversion to virulence. Therefore, the development of effective and safe vaccines against chicken coccidiosis is still urgently needed.

**Methods:**

In this study, a novel oral vaccine against *Eimeria tenella* was developed by constructing a recombinant *Lactobacillus plantarum* (NC8) strain expressing the *E. tenella* RON2 protein. We administered recombinant *L. plantarum* orally at 3, 4 and 5 days of age and again at 17, 18 and 19 days of age. Meanwhile, each chick in the commercial vaccine group was immunized with 3 × 10^2^ live oocysts of coccidia. A total of 5 × 10^4^ sporulated oocysts of *E. tenella* were inoculated in each chicken at 30 days. Then, the immunoprotection effect was evaluated after *E. tenella* infection.

**Results:**

The results showed that the proportion of CD4^+^ and CD8^+^ T cells, the proliferative ability of spleen lymphocytes, inflammatory cytokine levels and specific antibody titers of chicks immunized with recombinant *L. plantarum* were significantly increased (*P* < 0.05). The relative body weight gains were increased and the number of oocysts per gram (OPG) was decreased after *E. tenella* challenge. Moreover, the lesion scores and histopathological cecum sections showed that recombinant *L. plantarum* can significantly relieve pathological damage in the cecum. The ACI was 170.89 in the recombinant *L. plantarum* group, which was higher than the 150.14 in the commercial vaccine group.

**Conclusions:**

These above results indicate that *L. plantarum* expressing RON2 improved humoral and cellular immunity and enhanced immunoprotection against *E. tenella*. The protective efficacy was superior to that of vaccination with the commercial live oocyst vaccine. This study suggests that recombinant *L. plantarum* expressing the RON2 protein provides a promising strategy for vaccine development against coccidiosis.

**Graphical Abstract:**

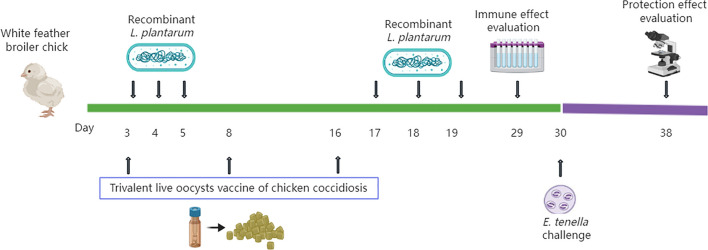

## Background

Chicken coccidiosis, a protozoan disease of the intestinal epithelial cells of chickens mainly caused by *Eimeria tenella*, seriously threatens the growth and development of chicks and causes serious economic losses to the poultry industry. It is one of the most serious parasitic diseases affecting chickens worldwide [1, 2]. The commonly used method for the prevention and treatment of this disease is still chemical drugs based on anticoccidials. Public concern about drug residues in broiler chicks due to the excessive use of anticoccidials and the emergence of drug-resistant strains of *Eimeria *spp. have affected the use of traditional drugs [3, 4]. Live oocyst vaccination is currently an alternative to anticoccidials for the prevention of coccidiosis. However, the use of this type of vaccine has several drawbacks, such as poor immunological protection and potential reversion to virulence [5, 6]. Therefore, it is important to develop a new, safe and environmentally friendly vaccine. Currently, the emerging genetically engineered vaccine compared with other methods is less time-consuming and costly to produce and easier to apply. The development of genetically engineered vaccine has become a rapidly growing field in anticoccidial treatment.

*Lactobacillus plantarum* is recognized as a safe or GRAS (generally recognized as safe) [7], noninvasive lactic acid bacteria (LAB) with good adhesion and immunogenicity, and it is a probiotic with immunomodulatory function. The use of *L. plantarum* has become an attractive strategy for the development of novel vaccines by using it as a recombinant vector carrying antigen, and some studies have shown that *L. plantarum* also has anticoccidial properties [8]. Poly-γ-glutamic acid synthetase A (pgsA’) is a component of the *Bacillus subtilis* polyglutamate synthetase system, which anchors exogenous proteins to the surface of the bacterium and is often used as a surface display element. Currently, pgsA’ has been confirmed to be anchored on the surface of *L. plantarum* and can trigger an extremely strong mucosal immune response [9]. Dendritic cells (DCs) are the most powerful specialized antigen-presenting cells (APCs) in organisms, and they are widely distributed in the gastrointestinal epithelium, serving as a bridge between natural and acquired immunity [10]. In a previous study, it was shown that the combination of dendritic cell-targeting peptide (DCpep) and protective antigen, delivered by LAB, significantly improved the systemic immune response triggered by the antigen [11].

*Eimeria tenella* belongs to the apicomplexan protozoans, and its rhoptry neck 2 (RON2) protein interacts with apical membrane antigen 1 protein (AMA1) to form the moving junction, which plays a key role in the invasion of host cells by *E. tenella*. The RON2 protein is an important invasion-associated molecule expressed during the infestation stage of *E. tenella*, and it is able to make the host to produce specific antibodies [12]. In recent years, studies have shown that the RON2 protein has good immunogenicity, and there have been many reports on the use of rhoptry neck protein antigens as vaccine candidates for preventing and treating diseases caused by other parasites, such as *Babesia bovis* and *Toxoplasma gondii* [13, 14]. A previous study indicated that truncated rhoptry neck protein 2, especially its N-terminal fragment (rN-BmRON2), plays an important role in the invasion of host red blood cells, confers immune protection and shows good potential as a candidate vaccine against babesiosis [15]. However, novel anticoccidial vaccines based on the RON2 protein have rarely been reported.

In this study, recombinant *L. plantarum* NC8-pSIP-409-pgsA'-RON2-Dcpep and NC8-pSIP-409-pgsA'-RON2 were successfully constructed by using *L. plantarum* NC8 as a vaccine vector, pSIP-409-pgsA’ as an expression vector, RON2 protein as a protective antigen and Dcpep as an adjuvant. The chicks were immunized with recombinant *L. plantarum*, and the immunoprotection effect was evaluated in a chicken *E. tenella*-infected model. The results may help develop a novel strategy for the prevention and control of *E. tenella*.

## Methods

### Parasite, plasmid, and fragment

The *E. tenella* strain was maintained at 4 °C in a 2.5% potassium dichromate solution in our laboratory and passed at least three times a year by chickens. The details of the strains, plasmids and primers used in this study are presented in Table 1. *Lactobacillus plantarum* strain NC8 was grown in MRS medium without agitation at 30 °C, and *Escherichia coli* strain DH5α was grown in LB medium.

**Table 1:** Strains, plasmids and primers used in this study

Plasmids or strains or primers	Description	Source
Strains pUC57- RON2-Dcpep	Synthesized RON2-Dcpep gene	Sangon Biotech Co., Ltd. (Shanghai), China
pSIP409-pgsA’	PsppIP, sppR, K, pgsA’, Em^r^	Laboratory Construction
*Escherichiacoli* DH5α	Host strain	TaKaRa Corporation, Japan
*Lactobacillus plantarum* NC8	Host strain, plasmid-free, silage isolate	Laboratory Source
Primers		
RON2-F-XbaI RON2-R-HindIII	GCTCTAGAATGAGGAGCGCCGGCTTCC CCAAGCTTTTAATGGTGATGGTGATGATGGAAATCT	Sangon Biotech Co., Ltd. (Shanghai), China Sangon Biotech Co., Ltd. (Shanghai), China

### Construction of recombinant *L. plantarum* NC8-pSIP-409-pgsA’-RON2-Dcpep

A codon-optimized RON2-Dcpep gene was optimized and synthesized by Sangon Biotech Co., Ltd. (Shanghai), according to the C-terminal extracellular region (aa.1082–1402) of RON2 (GenBank: XM_013375678). The *XbaI* site was added at the 5ʹ end, the *HindIII* site and the Flag sequence were added at the 3ʹ end, and the plasmid PUC-57-RON2-Dcpep was obtained. In addition, plasmid DNA was extracted for *HindIII* and *XbaI* double digestion, and RON2-Dcpep gene fragments were recovered. The RON2 gene was amplified using plasmid PUC-57-RON2-Dcpep as a template with a pair of PCR primers, RON2-F-XbaI (GCTCTAGAATGAGGAGCGCCGGCTTCC) and RON2-R-HindIII (CCAAGCTTTTAATGGTGATGGTGATGATGGAAATCT); the primer pairs for amplification of target genes are presented in Table 1. The plasmid RON2 and plasmid RON2-Dcpep were ligated into the vector pSIP-409-pgsA’ using T4 ligase, respectively. The prepared pSIP-409-pgsA’-RON2 and pSIP-409-pgsA’-RON2-Dcpep vectors were transformed into *E. coli* DH5α competent cells. These plasmids were verified by sequencing at Sangon Biotech Co., Ltd. (Shanghai). Subsequently, these plasmids were extracted and transformed into *L. plantarum* NC8 via electroporation. The recombinant *L. plantarum* strains were named NC8-pSIP-409-pgsA’-RON2 and NC8-pSIP-409-pgsA’-RON2-Dcpep, respectively. *Lactobacillus plantarum* NC8-pSIP-409-pgsA’ and two recombinant *L. plantarum* strains were anaerobically cultured at 37 °C in MRS medium. The optical density (OD) value of cultured bacteria was measured at 600 nm every 2 h to establish the 24 h growth curve of each bacterial strain.

### Western blotting

Recombinant *L. plantarum* was cultured at 30 °C without shaking in MRS broth supplemented with 10 mg/ml erythromycin. When the OD600 reached 0.3, 50 ng/ml, sakacin P-inducing peptide (SppIP) was added to induce antigen expression. After 10 h of induction at 30 °C, recombinant *L. plantarum* was collected, and the target protein was obtained by repeated freeze-thaw lysis at − 80 °C. Proteins were separated by SDS-PAGE on 10% acrylamide gels and transferred to PVDF membranes. The membranes were blocked with 5% skim milk powder for 1 h at room temperature, and RON2-Dcpep and RON2 proteins were detected by anti-flag Tag (1:10,000) (Sigma, Saint Louis, MO) monoclonal antibody and anti-his Tag (1:10,000) (Sigma) monoclonal antibody, respectively. The secondary antibody was horseradish peroxidase (HRP)-labeled goat anti-mouse IgG (1:100).

### Challenge and immunization procedures

White feather broiler chicks (1 day old) were raised in a formaldehyde-fumigated animal house with dry heat-sterilized rear utensils, drank high-temperature sterilized distilled water and were fed pellets without any anticoccidials, strictly guaranteed to be free from *E. tenella* contamination. In this study, 120 chicks were divided into six groups (*n* = 20) (Table 2), and the specific grouping immunization procedures are shown in Table 2. Chicks were immunized with recombinant *L. plantarum* (1 × 10^9^ CFU per chicken), vaccinated orally via gavage at 3 to 5 days of age, and a booster vaccination was administered at 17 to 19 days of age using the same quantity of recombinant *L. plantarum*. Meanwhile, each chick in the commercial vaccine group was orally immunized with 3 × 10^2^ trivalent live oocyst vaccine of chicken coccidiosis according to the manufacturer’s instructions (SKYSTAR BIO-PHARM Co., Ltd.) at 3, 8 and 16 days. A total of 5 × 10^4^ sporulated oocysts of *E. tenella* were inoculated into all chickens in each group (RON2 group, RON2-Dcpep group, empty vector group, challenge group and vaccine group) except the PBS group at 30 days. Then, ten chickens of each group were killed to collect the samples at 29 and 37 days.

**Table 2:** Experimental design and immunization procedures

Groups	Animals	Oral immunization dose	Challenge with *Eimeria tenella* sporulated oocysts
PBS	20	200 μl PBS	Unchallenged
RON2	20	200 μl NC8(pSIP409-pgsA’-RON2)/1.0 × 10^9^ CFU/chicken	5.0 × 10^4^/chicken
RON2-Dcpep	20	200 μl NC8(pSIP409-pgsA’-RON2-Dcpep)/1.0 × 10^9^ CFU/chicken	5.0 × 10^4^/chicken
Empty vector	20	200 μl NC8(pSIP409-pgsA’)/1.0 × 10^9^ CFU/chicken	5.0 × 10^4^/chicken
Challenge	20	200 μl PBS	5.0 × 10^4^/chicken
Vaccine	20	200 μl/3.0 × 10^2^ live oocysts of coccidia/chicken	5.0 × 10^4^/chicken

### Splenic lymphocyte proliferative capacity assay

A CCK-8 kit (Invitrogen, CA) was used to detect cell proliferation. To collect the samples, ten chickens of each group were killed at 29 days by cervical dislocation. Single-cell suspensions of lymphocytes were extracted from fresh spleen tissue using a spleen lymphocyte separation kit (Solarbio, Beijing, China). Single-cell suspensions from each group were inoculated into 96-well plates (5 × 10^5^ cells/well). Spleen cells were stimulated with RON2 protein (5 mg/ml) at 41 °C with 5% CO_2_ for 48 h. The stimulation index (SI) was calculated according to the formula SI = (OD test group-OD PBS control)/(OD *E. tenella* control-OD PBS control).

### ELISA

The levels of sIgA antibodies in intestinal lavage fluid and the levels of specific IgG antibodies and the cytokines IFN-γ and IL-2 in serum were measured by ELISA at 29 and 38 days. Briefly, five chickens were randomly selected from each group for necropsy. The duodenum was rinsed with PBS and an appropriate amount of PMSF was added. After shaking evenly, it was centrifuged at 4000 rpm for 15 min, and the supernatant was suctioned and stored at − 80 °C. Blood collected by cardiac puncture was centrifuged, and serum was collected and stored at − 80 °C. Then, the commercial ELISA kit from Jiangsu Kote Biotechnology Co., Ltd., was used for detection.

### Flow cytometry

The spleen cell suspensions were prepared as mentioned above at 29 days, and flow cytometry was performed to measure the percentages of CD3^+^CD4^+^ and CD3^+^CD8^+^ T cells as previously described [16]. Briefly, the isolated cells (1 × 10^6^/group) were incubated with mouse anti-chicken CD3-FITC, mouse anti-chicken CD4-APC and mouse anti-chicken CD8a-PE (BD Bioscience, USA). The samples were quantified by flow cytometry (BD LSRFortessa™, USA), and the data were analyzed using FlowJo.

### Anticoccidial index

After *E. tenella* challenge, body weight was recorded every day. At 36 to 38 days, fecal samples were collected for oocysts per gram (OPG) count examination employing the modified McMaster technique [17]. Then, the oocyst value (0–40) was quantified based on the oocyst ratio [18]. The oocyst ratio was calculated for each group according to the following formulae. The oocysts ratio (%) = (the OPG in the experiment/the OPG in the challenge) × 100%. The animals were killed at 38 days, and cecum samples from each group were collected. The cecal lesions of chickens in each group were scored on a scale from 0 to 4 according to the method previously described by Johnson and Reid [19]. Lesion score = the average lesion score in each group × 10. Then, the samples were fixed in 10% buffered formalin for 24 h, followed by paraffin embedding and standard H&E staining as described previously [20]. The anticoccidial index (ACI) was calculated by the number of OPG in feces, body weight and cecal lesions, which was evaluated as an immunoprotection after challenge with *E. tenella*. The calculation formula was as follows: ACI = (relative rate of weight gain + survival rate) − (lesion value + oocyst value). The ACI values are quantified according to the following criteria: ACI < 120, a mild curative effect; 120 < ACI ≤ 160, a moderate curative effect; 160 < ACI ≤ 180, a marked curative effect; and ACI > 180, an excellent curative effect [21].

### Statistical analysis of data

Graphs were generated using Graph Prism 9.0 software, and data statistics were performed and analyzed using Graph Prism 9.0, expressed as the mean ± SEM. Differences were compared using one-way ANOVA (*P < 0.05; **P < 0.01; ***P < 0.001).

## Results

### Construction and expression analysis of recombinant plasmids

**Fig. 1:**
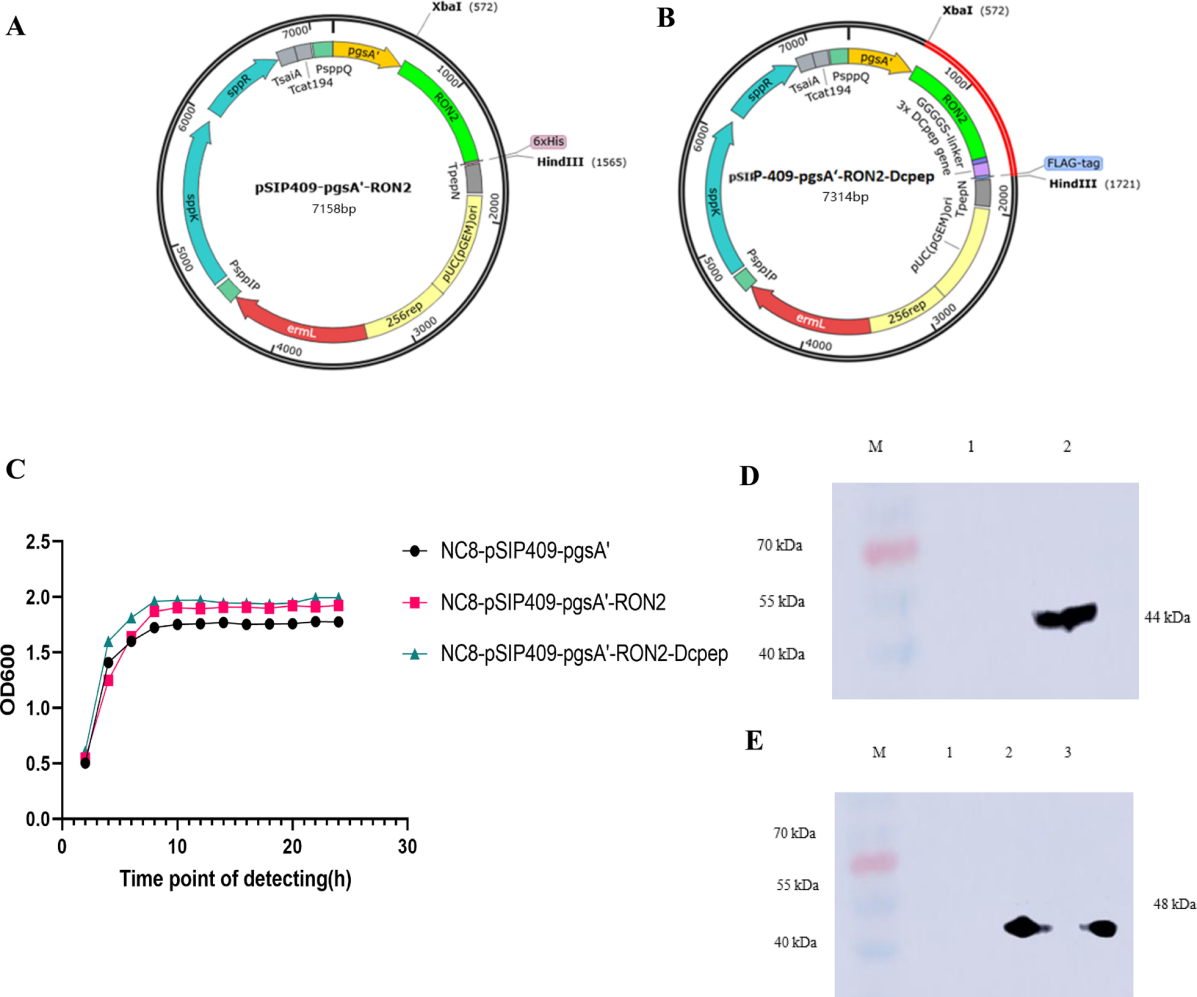
Synthesis of pSIP-409-pgsA;-RON2 and pSIP-409-pgsA;-RON2-Dcpep on *Lactobacillus plantarum*. **A** Recombinant pSIP-409-pgsA’-RON2 plasmid mapping. **B** Recombinant pSIP-409-pgsA’-RON2-Dcpep plasmid mapping. **C** Growth curves of the three strains. **D** Western blot detection of the pSIP-409-pgsA’-RON2 gene expressed by NC8-pSIP-409-pgsA’-RON2 (kDa). M: protein marker. Lane 1: NC8-pSIP409-pgsA’, Lane 2: NC8- pSIP-409-pgsA’-RON2. **E** Western blot detection of the pSIP-409-pgsA’-RON2-Dcpep gene expressed by NC8-pSIP-409-pgsA’-RON2-Dcpep (kDa). M: protein marker. Lane 1: NC8-pSIP409-pgsA’, Lanes 2 and 3: NC8-pSIP-409-pgsA’-RON2-Dcpep

Recombinant plasmids pSIP-409-pgsA’-RON2 and pSIP-409-pgsA’-RON2-Dcpep were successfully constructed, as shown in Fig. 1A and B, respectively. The growth curves showed that the recombinant *L. plantarum* RON2, RON2-Dcpep and the empty vector could grow normally in MRS medium and reached a growth peak at 10 h (Fig. 1C). Confirmation was performed by Western blotting using Flag tag and His tag specific antibodies. RON2 was detected at 44 kDa (Fig. 1D), and RON2-Dcpep was detected at 48 kDa (Fig. 1E).

### Cytokine IFN-γ and IL-2 were induced in immunized chicks

**Fig. 2:**
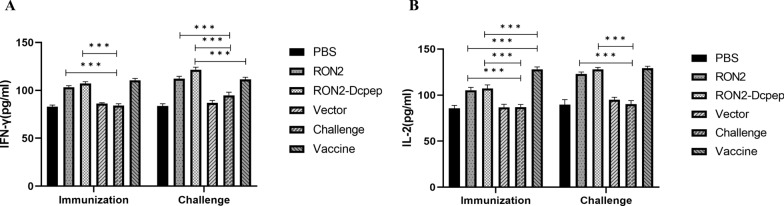
Levels of cytokines in the serum of immunized chicks. Measurement of IFN-γ levels (**A**) and IL-2 levels (**B**) in the serum by ELISA of chicks at 29 and 38 days. The data shown represent the mean ± SE (*n* = 5), which were compared by one-way ANOVA (*P < 0.05, **P < 0.01 and ***P < 0.001)

The production of inflammatory cytokines in serum after secondary immunization and after *E. tenella* challenge was also determined by ELISA. As shown in Fig. 2A and B, the expression levels of IFN-γ and IL-2 in the recombinant *L. plantarum* group were significantly higher than those in the challenge group after secondary immunization (*P* < 0.001) (ANOVA; *F*_IFN-γ_ = 304.2, *F*_IL-2_ = 141.7; *P* < 0.0001). The results presented higher levels of IFN-γ and IL-2 in the RON2 and RON2-Dcpep groups after *E. tenella* challenge; compared with those in commercial vaccine group, the levels of IFN-γ were markedly increased in the RON2-Dcpep group (*P* < 0.001) (ANOVA; *F*_IFN-γ_ = 183.1, *F*_IL-2_ = 174.1; *P* < 0.0001) (Fig. 2A).

### Specific IgG and sIgA antibody levels were enhanced by recombinant *L. plantarum*

**Fig. 3:**
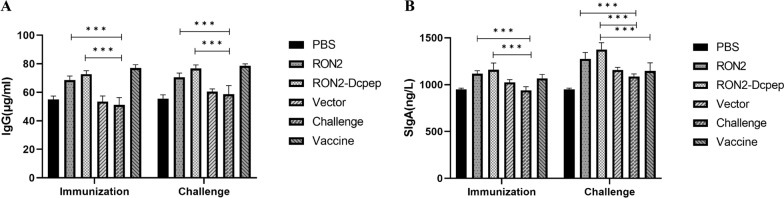
Levels of IgG and sIgA antibodies of immunized chicks. Measurement of IgG levels in the serum (**A**) and sIgA levels in the intestinal lavage (**B**) by ELISA of chicks at 29 and 38 days. The data shown represent the mean ± SE (*n* = 5), compared by one-way ANOVA (*P < 0.05, **P < 0.01 and ***P < 0.001)

The levels of humoral immunity were examined using ELISA before and after *E. tenella* challenge. In general, immunization with recombinant *L. plantarum* RON2-Dcpep (*P* < 0.01) and recombinant *L. plantarum* RON2 (*P* < 0.01) (ANOVA; *F*_IgG_ = 51.32, *F*_sIgA_ = 17.55; *P* < 0.0001) significantly increased the production of IgG in serum and sIgA in intestinal samples compared to other groups (Fig. 3A and B). The levels of specific IgG and sIgA antibodies were higher in the RON2-Dcpep group than in the RON2 group, but the differences were not significant.

### Spleen lymphocyte proliferation in immunized chicks was enhanced

**Fig. 4:**
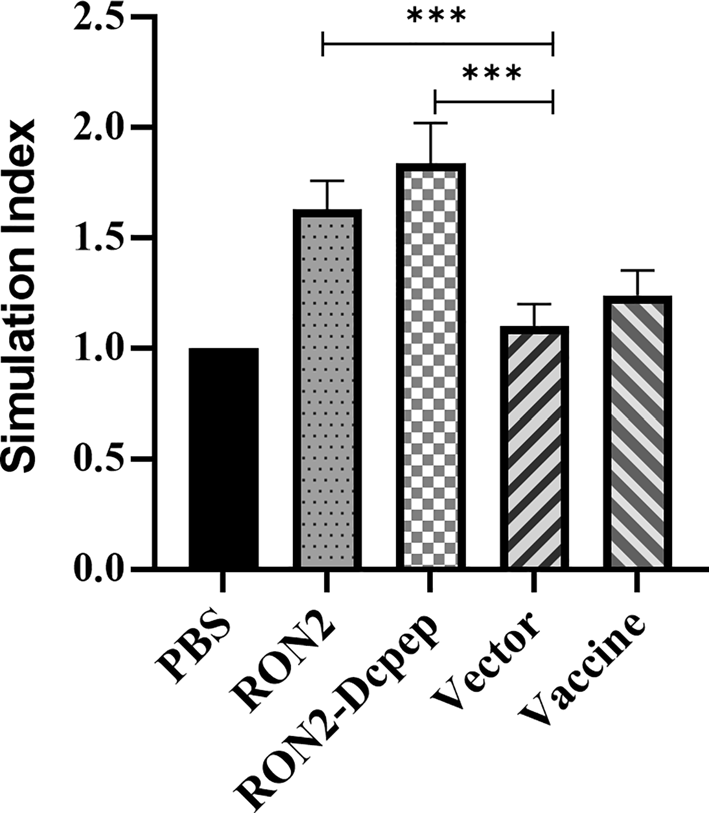
Proliferation ability of spleen lymphocytes stimulated by RON2 protein was enhanced. Evaluation of cell proliferation status by the CCK-8 assay. The data shown represent the mean ± SE (*n* = 5), compared by one-way ANOVA (*P < 0.05, **P < 0.01 and ***P < 0.001)

To investigate the effect of recombinant *L. plantarum* on immune function in chicks, the proliferation of spleen lymphocytes stimulated by recombinant RON2 protein was evaluated, and the corresponding stimulation index (SI) was calculated. The proliferative ability of spleen lymphocytes was significantly increased in the chicks immunized with recombinant *L. plantarum* (*P* < 0.001) (ANOVA; *F* = 44.08; *P* < 0.0001) in contrast to that of the other groups (Fig. 4).

### Recombinant *L. plantarum* stimulated the differentiation of T cells

**Fig. 5:**
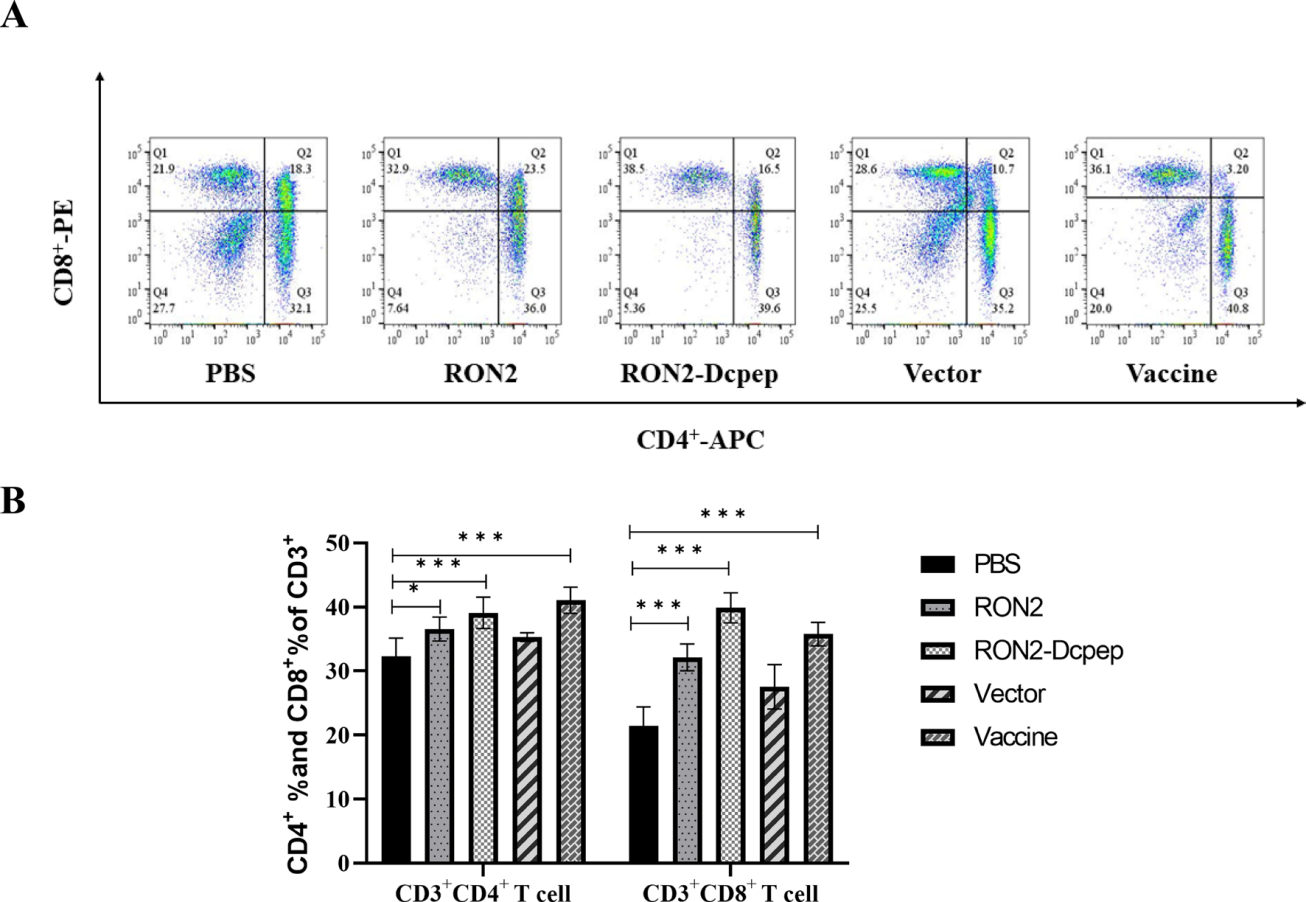
Recombinant *Lactobacillus plantarum* induced a robust ratio increase in CD3^+^CD4^+^ and CD3^+^CD8^+^ T cells. The percentage of CD3^+^CD4^+^ and CD3^+^CD8^+^ T cells was determined at 30 days by FACS. **A** Panels representing CD3^+^CD4^+^ and CD3^+^CD8^+^ T cells for each group. **B** Percentages of CD3^+^CD4^+^ T cells and CD3^+^CD8 ^+^ T cells among spleen cells were detected using flow cytometry. The data shown represent the mean ± SE (*n* = 5), compared by one-way ANOVA (*P < 0.05, **P < 0.01 and ***P < 0.001)

The percentages of CD3^+^CD4^+^ and CD3^+^CD8^+^ T cells in spleen lymphocytes were determined by FACS after immunization with recombinant *L. plantarum*, and the results are shown in Fig. 5A and B. The proportions of CD3^+^CD4^+^ T cells and CD3^+^CD8^+^ T cells were significantly increased in the RON2-Dcpep and RON2 groups compared with those in PBS group (*P* < 0.001) (ANOVA; *F*_CD3+CD4+ T cell_ = 12.92, *F*_CD3+CD8+ T cell_ = 37.75; *P* < 0.0001). However, the difference between the RON2-Dcpep group and RON2 group was not significant.

### Recombinant *L. plantarum* reduced cecal damage

**Fig. 6:**
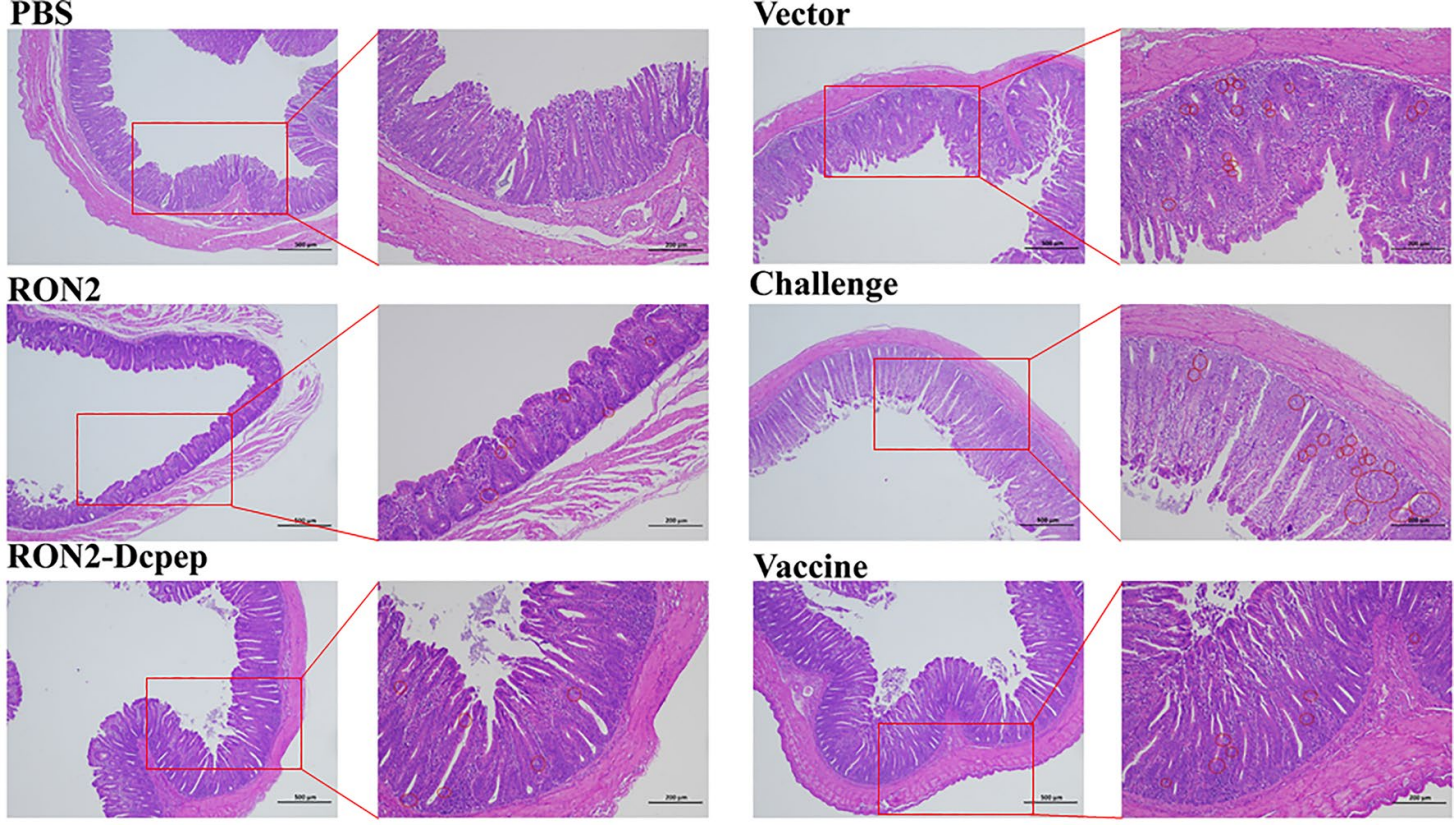
Histopathological evaluation of the cecum after challenge. Sample sections were stained using H&E (× 40, × 100 magnification). The cecal sections of each experiment group is magnified at right. The oocysts are circled by the red circles

The cecal specimens were stained with H&E to examine histopathological changes. As shown in Fig. 6, in the challenge group and vector group, the intestinal villous structure was severely damaged, the number of red blood cells and inflammatory cells was increased, and a large number of coccidian oocysts accumulated in the cecum lumen. The RON2 and RON2-Dcpep group exhibited no significant pathological changes in the intestinal tissues, the structures of the cecal tissues were relatively complete, and the number of oocysts in the cecum cavity was significantly reduced.

### Anti-coccidial index

**Fig. 7:**
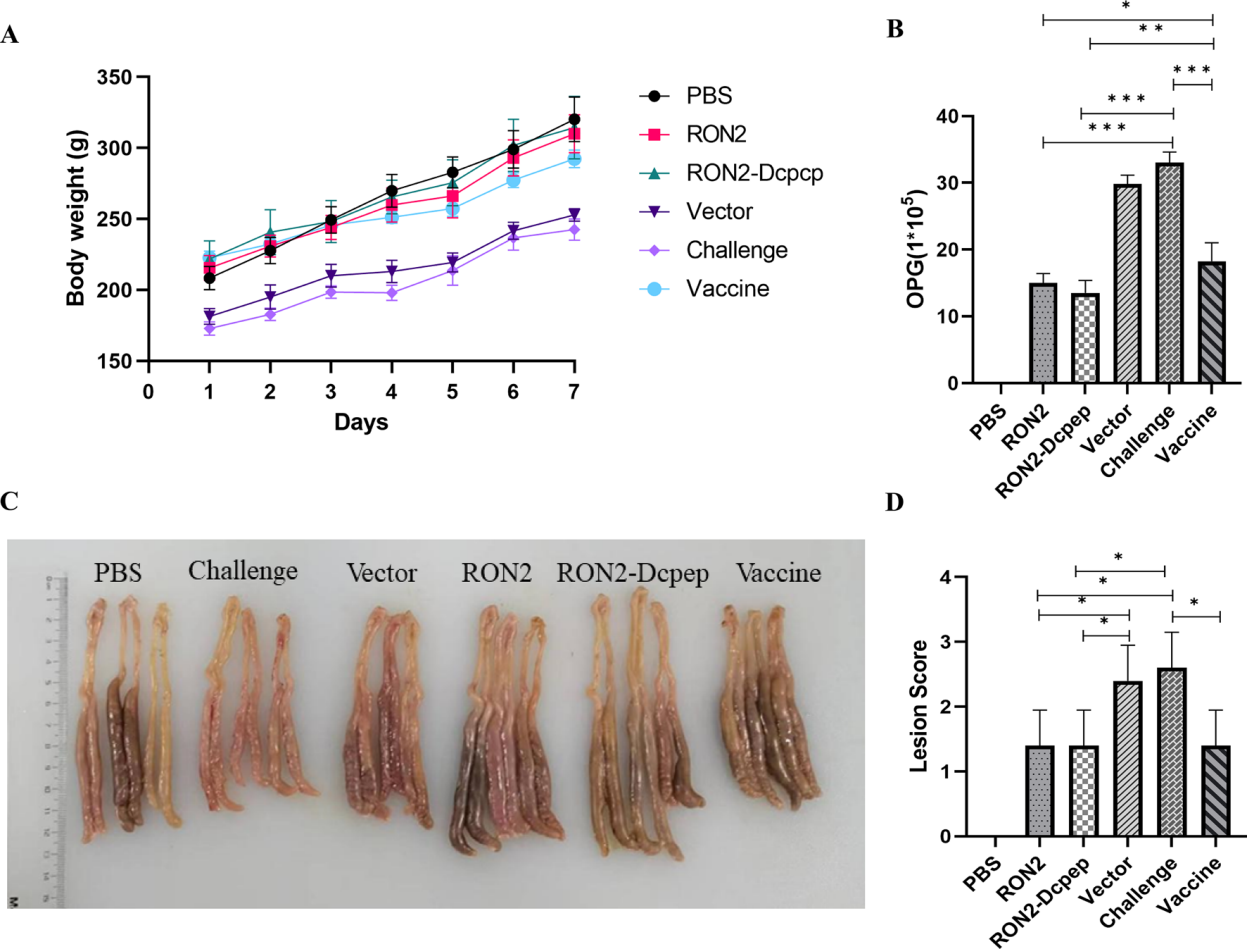
Protective efficacy of recombinant *Lactobacillus plantarum* against *Eimeria tenella* challenge in chicks. **A** The overall body weight of chicks after *E. tenella* challenge. **B** Statistics of OPG values. Intestinal macroscopic lesions (**C**) and lesion scores (**D**) were used to evaluate coccidiosis severity. The data shown represent the mean ± SE (*n* = 5), compared by one-way ANOVA (*P < 0.05, **P < 0.01 and ***P < 0.001)

The anti-coccidial index (ACI) is an important indicator for evaluating the anticoccidial activity of vaccines. To investigate whether the immunological response induced by RON2 provides protection against *E. tenella* infection, ACI, which is a protective immune index after challenge with *E. tenella*, was calculated by the number of OPG in feces, body weight and cecal lesions, and the results are shown in Table 3. Briefly, the body weight and number of OPG in chickens were observed, and lesion scores in the cecum were recorded after challenge. As shown in Fig. 7A, the overall body weight of the recombinant *L. plantarum* group was higher than that of the commercial vaccine group. The number of OPG after challenge is shown in Fig. 7B, which shows that the recombinant *L. plantarum* group was significantly lower than the challenge and commercial vaccine groups (*P* < 0.05) (ANOVA; *F* = 17.17; *P* < 0.0001). The cecum lesions were scored at 7 days after *E. tenella* challenge. The lesion score was significantly decreased in the RON2-Dcpep and RON2 groups compared with that in the commercial vaccine group (*P* < 0.05) (ANOVA; *F* = 298.1; *P* < 0.0001) (Fig. 7C and D). Therefore, the RON2 and RON2-Dcpep group (170.89) showed the highest ACI value, while the ACI value of the challenge group (75.37) was the lowest (Table 3). The ACI value of the RON2 and RON2-Dcpep groups was 170.49 and 170.89 and showed a marked curative effect against *E. tenella*. The ACI value of the commercial vaccine group was 150.14 and showed a moderate curative effect. Interestingly, the immunoprotection against *E. tenella* in chickens was better in the RON2 and RON2-Dcpep groups compared to the commercial vaccine group.

**Table 3:** Protective effects in each group

Groups	Relative body weight gain (%)	Survival rate (%)	Oocysts value	Lesion scores	ACI
PBS	100.0	100	0	0	200.00
RON2	93.5	100	10	13	170.49
RON2-Dcpep	93. 9	100	10	13	170.89
Vector	58.4	100	40	21	97.39
Challenge	57.4	80	40	22	75.37
Vaccine	84.1	100	20	14	150.14

## Discussion

The development of novel vaccines for chicken coccidiosis has become a novel strategy, but one of the challenges is that these vaccines are disrupted by the low pH of the gastric environment. One promising solution is the use of *L. plantarum* as an oral vaccine delivery system. *Lactobacillus plantarum* is often considered safe or “GRAS,” and most of these bacteria are naturally acid tolerant at low pH, allowing them to survive effectively in the harsh low-acid environment of the stomach and complex intestinal conditions [22–24]. Therefore, *L. plantarum* is a highly efficient and potential mucosal antigen delivery vehicle. Andrew et al. demonstrated that vaccination with a recombinant *L. plantarum* strain vaccine could increase sIgA antibodies in the intestine and IgG antibodies in the serum in chickens [25]. Anchored expression of exogenous proteins on the surface of LAB has been shown to be effective in inducing protective immune responses in chickens [26]. The anchored expression vector polyglutamate synthase A (pgsA) antigen delivery system used in this study has been shown to induce a better protective immune response than secretory expression vectors and cytoplasmic expression vectors [27].

*Eimeria tenella* is an intracellular protozoan parasite, which belongs to the *Apicomplexa phylum* [28]. The RON2 protein is an essential invasion-associated molecule expressed during the infestation stage of *Apicomplexa* protozoan and plays an important role in regulating immune response of the host and preventing infection [29–31]. Therefore, in this study, recombinant *L. plantarum* NC8 containing the *E. tenella* RON2 protein was constructed, and the immune efficacy of the recombinant *L. plantarum* was further evaluated in a chicken *E. tenella*-infected model. The animal experimental results demonstrated that chickens immunized with *L. plantarum* expressing RON2 protein displayed higher body weight gain, lower cecal lesion scores and lower oocysts value compared to the vector group. Histopathological examination also showed that the *E. tenella*-induced pathological damage of chicken cecum was markedly reduced by the immunization with recombinant *L. plantarum*. The above results indicate that the chickens showed a higher ACI value and marked effective protection against *E. tenella* after oral immunization with recombinant *L. plantarum*.

DCpep is highly targeted to DCs that normally express antigens to enhance the efficacy of the vaccine. A previous study showed that Dcpep expression in LAB could enhance the uptake of the target antigen by intestinal DCs, produce stronger specific immune responses and therefore provide more efficient protection against pathogens [32]. Chen et al. showed that Dcpep expression in LAB could significantly enhance antibody levels [33]. Therefore, in the present study, the surface expression system of *L. plantarum* NC8-pSIP-409-pgsA' was further modified by fusing DCpep to *E. tenella* RON2 protein encoding gene, and the recombinant *L. plantarum* NC8-pSIP-409-pgsA'-RON2-Dcpep displaying surface-anchored DCpep-RON2 fusion protein was constructed with expectation of enhancing the immunogenicity of target RON2 protein. The chickens in RON2 and RON2-Dcpep group exhibited high levels of antibody secretion before and after *E. tenella* challenge (*P* < 0.01). Although the levels of IgG and sIgA antibodies of chickens in RON2-Dcpep group were slightly higher than in RON2 group, the difference was not significant. The ACI value of the RON2-Dcpep group was 170.89, slightly higher than that of RON2 group (170.49); both showed a marked curative effect against *E. tenella*. These results indicated that Dcpep might enhance mucosal immunity mediated by sIgA and IgG to improve humoral immunity of chicks after *E. tenella* challenge. This finding suggests that Dcpep has good potential as a mucosal vaccine adjuvant.

During *E. tenella* infection, immune defense against the parasite was strongly induced in chickens, and cell-mediated immune response plays a key role in this defense [34]. The proportions of CD4^+^ T cells and CD8^+^ T cells increased significantly after immunization with recombinant *L. plantarum* (*P* < 0.01). T cells play a major protective role in immunization against *E. tenella*. Studies have shown that Th1 type cytokines play a major role in host resistance to *E. tenella* infection [35]. Th1 cells enhance cellular immunity by releasing cytokines and recruiting monocytes/macrophages and lymphocytes [36, 37]. In this study, the results showed that immunization with recombinant *L. plantarum* strains resulted in a significant increase in the serum expression levels of IFN-γ and IL-2 cytokines, and the expression of IFN-γ was significantly higher in the RON2-Dcpep group than in the commercial vaccine group.

The production of IFN-γ in chickens is regarded as a hallmark of cell-mediated immunity against *E. tenella* infection, because it stimulates macrophages and other cells to produce free radicals that kill the parasite or host cells [38]. The crucial roles of IL-2 in resisting *E. tenella* infection were reported by Lillehoj [39]. IL-2 plays an important role in the immune system, primarily via its direct effects on T cells. It also promotes the differentiation of T cells to effector T cells and memory T cells. IL-2 has previously been used as an adjuvant component of the *E. tenella* DNA vaccine [40, 41].

## Conclusions

The present results demonstrate that fusion proteins were successfully anchored on the surface of *L. plantarum*. A clear enhancement of both cellular and humoral immunity was observed after immunization with recombinant *L. plantarum*. The production of proinflammatory cytokines increased significantly, the specific antibody levels were significantly increased, and the proportions of CD3^+^CD4^+^ T cells and CD3^+^CD8^+^ T cells were effectively increased. At the same time, these activated immune responses were accompanied by a significantly increased ACI. Recombinant *L. plantarum* had effective protection against *E. tenella* and could serve as a potential oral immunization strategy for chicks. In addition, these results support Dcpep as an effective adjuvant that has great potential for mucosal vaccines. This study provides new insights into the development of future vaccines against coccidiosis.

## Data Availability

The data are presented within the paper. Additional raw data are available on request from the corresponding author.

## Abbreviations

*E. tenella*: *Eimeria tenella*
ELISA: Enzyme-linked immunosorbent assay
ANOVA: Analysis of variance
Dcpep: Dendritic cell-targeting peptide
RON2: Rhoptry neck 2
IgG: Immunoglobulin G
sIgA: Secretory immunoglobulin A
IFN-γ: Interferon gamma
IL: Interleukin
ACI: Anticoccidial index
LAB: Lactic acid bacteria
GRAS: Generally recognized as safe
pgsA’: Poly-γ-glutamic acid synthetase A
DCs: Dendritic cells
APCs: Antigen-presenting cells
*E.coli*: *Escherichia coli*
SppIP: Sakacin P-inducing peptide
PVDF: Polyvinylidene difluoride
*L. plantarum*: *Lactobacillus plantarum*
CCK8: Cell counting kit-8
SI: Stimulation index
OPG: Oocysts per gram
MRS: De MAN, ROGOSA and SHARPE
